# Bioactivity of Aromatic Plant Hydrolates and Application for Pork Marination

**DOI:** 10.3390/antiox14121508

**Published:** 2025-12-16

**Authors:** Nieves González-Cantillo, María Jesús Martín-Mateos, Susana García-Torres, Jonathan Delgado-Adámez, Francisco María Vázquez, María Rosario Ramírez-Bernabé

**Affiliations:** Centro de Investigaciones Científicas y Tecnológicas de Extremadura (CICYTEX), Instituto Tecnológico Agroalimentario de Extremadura (INTAEX), Avda. Adolfo Suárez s/n, 06007 Badajoz, Spain; nieves.gonzalez@juntaex.es (N.G.-C.); mariajesus.martinmat@juntaex.es (M.J.M.-M.); susana.garciat@juntaex.es (S.G.-T.); jonathan.delgado@juntaex.es (J.D.-A.); francisco.vazquezp@juntaex.es (F.M.V.)

**Keywords:** hydrolate, marinate, essential oils, thymus, *Lavandula*, *Origanum*, antioxidant, antimicrobial, meat

## Abstract

The study evaluated the potential of *Thymus mastichina*, *Lavandula luisiery*, and *Origanum virens* hydrolates—essential oil-rich species found in the Dehesa ecosystem—as natural agents for pork preservation through marination. Hydrolates, the main by-products of essential oil distillation, were characterized by their volatile composition, total phenolic content, antioxidant capacity, and antimicrobial activity against *Escherichia coli* and *Listeria innocua*. Pork fillets were marinated (24 h) with increasing hydrolate concentrations (1:1, 1:1.5, 1:2 *w*/*w*) and then stored for eight days under refrigeration to assess microbial growth, oxidation, and color changes. All hydrolates exhibited similar antimicrobial effects, though *Origanum* showed the highest phenolic content and antioxidant activity. Marination had limited antimicrobial impact. Marination with *Origanum* and *Thymus* provided significant protection against lipid oxidation during storage, while *Lavandula* displayed limited or even pro-oxidative behavior at certain doses. Marination did not affect carbonyl formation although it caused slight changes in instrumental color. Overall, the study highlights the potential of *Lavandula*, *Thymus*, and *Origanum* hydrolates as sustainable alternatives to synthetic antioxidants in meat products, emphasizing their antioxidant—rather than antimicrobial—effect.

## 1. Introduction

The Dehesa constitutes an agrosilvopastoral system unique in the world, with great plant biodiversity, located in the southwest regions of Spain. Aromatic plants from the Dehesa produce essential oils, that are rich source of bioactive compounds. These essential oils are extracted from various plant materials, such as flowers, buds, seeds, leaves, twigs, bark, herbs, wood, fruits, and roots. The metabolites found in essential oils primarily include terpenes, terpenoids, phenylpropanoids, aldehydes, esters, alcohols, and ketones, whose bioactivity varies based on the structural configuration of the molecule. These compounds are secondary metabolites produced as part of the plant’s defense mechanism. According to existing studies, phenolic compounds are the main antibacterial agents contributing to the inhibition of microbial growth [1]. Essential oils from aromatic plants have been widely used in various industries due to their therapeutic properties and health benefits [2]. In the food industry, they have been utilized to extend the shelf life of products because of their antimicrobial properties, antifungal properties, and antioxidant properties.

Hydrolates are by-products of the distillation process used to extract essential oils from aromatic plants. Steam distillation is the most widely employed technique for essential oil extraction. Hydrolates are made up of condensed water from the distillation process, as well as polar, oxygenated, odor-imparting, hydrophilic, volatile oil constituents that form hydrogen bonds with water. They typically contain trace levels of essential oils (usually less than 1 g/L), along with a variety of water-soluble phytochemical constituents. Hydrolates are traditionally characterized by their dilute composition and relatively high acidity, with pH values generally ranging from 3.5 to 6.5. The aromatic profile of hydrolates is herbaceous and variable, fluctuating from mild to intense and from pleasant to unpleasant, occasionally resembling that of the corresponding essential oil depending on the specific distillation phase during which they are collected. As a readily obtainable by-product, hydrolates must be stored under aseptic conditions, in sealed containers, and maintained at low temperatures to preserve their integrity [3]. Although hydrolates are often discarded, strategies are being developed for the recovery of residual essential oils, such as liquid–liquid extraction or adsorption techniques, as these natural by-products have sparked increasing scientific and technological interest as potential natural antimicrobial agents [4]. Jakubczyk et al. [5] investigated the biological activities of hydrolates derived from various plants, including antimicrobial, antifungal, anti-inflammatory, and antioxidant properties.

The bioactivity of hydrolates are attributed to their main compounds and their functional groups, according to some researchers [6,7]. These activities are primarily driven by oxygenated, water-soluble compounds [8]. Moreover, Ni et al. [9] reported that the diverse biological activities of essential oils are primarily attributed to the chemical composition of their volatile constituents, many of which are also present in hydrolates, potentially explaining the similar bioactivities observed in these aqueous plant distillates. In recent years, the commercial utilization of hydrolates has expanded, particularly in cosmetic formulations and aromatherapy, where consumer demand for natural and safe ingredients has driven innovation. Moreover, these distillates and hydrolates are gaining recognition as versatile and valuable raw materials.

In line with circular economy principles, they offer sustainable strategies to valorize industrial by-products and mitigate environmental impacts caused by waste disposal. *Lavandula* spp., *Thymus* spp., and *Origanum* spp. are well-known medicinal and aromatic plants belonging to the *Lamiaceae* family. These species exhibit a wide range of biological activities, particularly notable antioxidant and antimicrobial effects, which have been demonstrated across various food matrices [10,11]. *Lavandula*, native to the Mediterranean basin, is a valuable source of phytochemicals, and its essential oils or extracts exhibit a wide range of pharmacological properties, including sedative, anti-inflammatory, antimicrobial, and antioxidant activities [12]. *Thymus* produces essential oils rich in terpenes, mainly monoterpenic phenols such as thymol and carvacrol [13]. *Origanum* spp., especially their leaves, are rich in terpenes and phenolic compounds, and their essential oils have shown in vitro antibacterial, antioxidant, and anti-inflammatory properties [14].

Meat and meat products require preservation strategies to prevent microbial spoilage and control deterioration reactions, primarily related to lipid and protein oxidation, which are considered the most important factors in preserving meat and meat products [15]. However, the use of preservatives in industrially processed foods is often perceived negatively by consumers, mainly due to concerns about potential toxicological effects and health risks. Consequently, the agri-food industry has developed new strategies to ensure microbiological safety and extended shelf life. In this regard, natural antioxidants or antimicrobials, which are typically low in toxicity and derived from natural, renewable, and sustainable sources, are considered a favorable alternative that can enhance both the quality and microbiological safety of foods [16].

Studies have shown that essential oils from plants can extend the shelf-life of food products and improve their safety by inhibiting microbial growth and oxidative processes. Although the use of essential oils in meat preservation is well-documented [17], research on the application of hydrolates for this purpose is less extensive. Industrial hydrolates exhibit low odor and flavor intensity due to their minimal essential oil content. Consequently, they could be incorporated into food products without significantly altering their sensory properties. This contrasts with the direct incorporation of essential oils into food formulations, which can negatively impact sensory quality and consumer acceptance [18]. High concentrations of essential oils may alter the sensory characteristics of food products due to their intense aroma and flavor. Furthermore, given their antimicrobial and antioxidant properties, hydrolates have the potential to be used as natural preservatives in the meat industry, warranting further investigation. Therefore, the main objective of this study is to characterize the bioactivity of hydrolates (obtained from the extraction process of essential oils) from *Lavandula luisiery*, *Thymus mastichina* and *Origanum virens* and to analyze their application for preservation of meat products through a marination process aimed at preventing microbial spoilage and oxidative degradation, thereby extending the shelf life of meat products.

## 2. Materials and Methods

### 2.1. Material

Three loins (m. *Longissimus dorsi*) from commercial pig breeds were acquired in a local market. Loins were manually sliced into 1 cm fillets which were homogeneously distributed among experimental groups (one slice from each loin was distributed in each batch). The pH of the meat was 5.3 ± 0.1. Regarding the proximate composition, the moisture content was 73.4 ± 0.5 g 100 g^−1^, the protein content was 23.1 ± 1.1 g 100 g^−1^ and the fat content was 2.1 ± 1.1 g 100 g^−1^. Three hydrolates were tested: *Lavandula luisiery*, *Thymus mastichina* and *Origanum virens*. These hydrolates were selected because their aromatic profiles are suitable for meat products, as the medicinal plants from which they are derived, have traditionally been used for meat preservation. All species were cultivated under experimental conditions in certified organic farming plots located at Finca La Orden (CICYTEX, Badajoz, Spain). The geographical coordinates of the cultivation sites were as follows: *Origanum virens* at 38°51′28″ N, 6°39′58″ W; *Lavandula luisiery* at 38°51′28″ N, 6°39′57″ W; and *Thymus mastichina* at 38°51′28″ N, 6°39′56″ W. The altitude for all locations was 193 m a.s.l.

Hydrolates were obtained using a 200 L distillation unit equipped (Pobel, Madrid, Spain) with both hydrodistillation and steam distillation options. The distillation method used was hydrodistillation in a Clevenger-type apparatus for 2 h. The extracted essential oil was stored in an amber vial at 4 °C. In all cases, hydrolates were produced by hydrodistillation. For each species, 25–35 kg of fresh plant material was immersed in 60 L of water and distilled for 2 h at 100–105 °C. The hydrolates were neither filtered nor sterilized after distillation; however, they were stored under refrigeration at 2–5 °C at all times. Yields varied depending on the species. The resulting hydrolates were stored in aseptic conditions at refrigeration temperature.

### 2.2. Experimental Design

Three fillets per batch were used to evaluate each concentration of hydrolates and were compared to control samples. Five independent fillets were used for the physicochemical characterization of the meat. A total of 65 fillets were analysed: 3 replicates × 10 batches (3 types of hydrolates × 3 doses + 1 control) × 2 storage times = 60 fillets + 5 fillets for meat characterization.

Three types of hydrolates were tested: *Lavandula luisiery*, *Thymus mastichina* and *Origanum virens*. Increasing meat-to-hydrolate ratios were evaluated for the marinated meat. Meat-to-hydrolate ratios of 1:1 (*w*/*w*, equal weights of meat and hydrolate), 1:1.5 (*w*/*w*), and 1:2 (*w*/*w*) were studied. Each loin fillet was individually marinated in the corresponding amount of hydrolate in a small plastic tray. The trays were sealed with commercial aluminium foil and stored under refrigeration at 5 °C for 24 h. The marinating time was selected based on preliminary tests and common industrial practices, ensuring adequate interaction between the hydrolates and the meat matrix. After marination, the hydrolate was removed in each fillet. Then, the fillets were individually vacuum packaged in plastic bags (OptiDure™ ODA7005, Cryovac, Madrid, Spain) with an oxygen permeability of 10 cm^3^/m^2^/24 h at 0% relative humidity. Vacuum packaging was carried out using Henkovac Proeco equipment (Henkovac International, Hertogenbosch, The Netherlands). Control group was vacuum packaged without being marinated (the 24 h before). All packaged samples were refrigerated at 5 °C for 8 days, a period chosen as it reflects a realistic refrigerated shelf-life for fresh meat. Samples were analyzed at day 1, after 24 h of marination (or 24 h of vacuum packaging in the case of control samples) and subsequent packaging; and at day 9 (after 8 days of storage). Colour and microbiological analysis were carried out in the fresh pork. Then the samples were stored at −80 °C until the rest analysis were carried out.

### 2.3. Hydrolates Characterization

#### 2.3.1. Volatile Profile Analysis

For the analysis of volatile compounds, 20 µL of each hydrolate were placed into a 20 mL vial (screw-capped with a Teflon–silicone septum). A 1 cm 50/30 μm DVB/CAR/PDMS SPME fiber (Supelco, Bellefonte, PA, USA) was utilized for the analysis of volatile compounds in the headspace of the previously prepared vial. The fiber was exposed to the headspace at 37 °C for 30 min. Volatile compounds were identified using an Agilent 6850 Series Gas Chromatography/Agilent 5975 Series Quadropole Mass Detector (Single) (Santa Clara, CA, USA) with a CombiPAL autosampler (CTC Analytics, Zwingen, Switzerland) and with an HP-5 capillary column (30 m × 0.32 mm × 0.25 μm; Agilent Technology, Santa Clara, CA, USA) was used. The injection port was at 230 °C, and the oven temperature was held at 35 °C for 10 min, increased to 250 °C (7 °C min^−1^), and held for 5 min, with a total running time of 45 min. The MS source and MS Quad temperatures were maintained at 230 °C, and 150 °C, respectively, scanning at 1 scan s^−1^ over a 40–300 *m*/*z* range. Identification of volatile compounds was achieved by comparing mass spectra with the NIST library (Agilent MSD ChemStation, E.02.01.1177 software) and by calculating the retention index using a series of n-alkanes. Some compounds were also confirmed by comparing their retention times with those of commercial standards (Sigma-Aldrich, St. Louis, MO, USA). Results are expressed as arbitrary area units (AAU) × 10^5^.

#### 2.3.2. Microbial Counts

10 g of sample—corresponding to the control fillets and those treated with hydrolates—was taken aseptically and homogenized with 90 mL of sterile peptone water in a laboratory blender (Stomacher R_ 400 Circulator) (Seward Limited, Worthing, UK) for 1 min. This initial mixture represented the first decimal dilution (10^−1^). From this suspension, serial decimal dilutions (10^−2^, 10^−3^, 10^−4^, etc.) were prepared using sterile peptone water, and 1 mL of each dilution was spread onto the corresponding culture media.

Mesophilic aerobic bacterial counts were performed using a standard PCA (Plate Count Agar) (Merck, 1.07881), and plates were incubated at 30 °C for 72 h. Molds and yeasts were incubated on Potato Dextrose Agar (Merck, Darmstadt, Germany) at 25 °C for 5 days. For *Clostridium* and *Bacillus*, dilution 1 was subjected to 80 °C for 10 min to eliminate vegetative forms and leave spores. *Clostridium* was seeded in TSC (Tryptose Sulfite Cycloserine) agar at 37 °C for 24 h anaerobically. Bacillus is surface-seeded in PCA and incubated aerobically at 27–28 °C for 24 h. The detection limit of the above techniques was 10 CFU g^−1^.

#### 2.3.3. Phenolic Compounds Content

The hydrolate was analyzed following the same procedure used for the phenolic extracts. Total phenolic content was determined according to Lima et al. [19]. In brief, 10 mL of Milli-Q water (Millipore, Burlington, MA, USA) and 0.5–1 mL of the hydrolate were added to 25 mL volumetric flasks. Subsequently, 1.3 mL of Folin–Ciocalteu reagent was added, the mixture was vortexed, and allowed to react for 3 min. Then, 2.5 mL of saturated sodium carbonate solution was added, and the volume was brought to 25 mL with Milli-Q water. The reaction proceeded in the dark for 1 h, after which absorbance was recorded at 760 nm. Gallic acid was used for the generation of a standard curve, and the results were expressed as milligrams of Gallic acid equivalent (GAE) per 100 g of sample weight on wet base (WB).

#### 2.3.4. Antioxidant Activity

Antioxidant activity (AA) was quantified using a modified ABTS ([2,2′-azinobis (3-ethylbenzothiazoline-6-sulphonic acid)]) + spectrophotometric assay adapted for microwell plates, following the method described by Turoli et al. [20], The ABTS^+^ radical cation was prepared by reacting 7 mM ABTS with 2 mM potassium persulfate, using acidified ethanol for the lipophilic assay and phosphate buffer (pH 7) for the hydrophilic assay. Both mixtures were kept in the dark at 4 °C for 12–16 h until an absorbance of 1.3–1.6 at 734 nm was reached. For the analysis, an aliquot of each extract (lipophilic or hydrophilic) was mixed with the corresponding ABTS^+^ working solution, and the absorbance at 734 nm was measured for at least 30 min using a microplate reader (Tecan Spark multimode microplate reader, Grödig, Austria). Trolox was used as the reference compound, and results were expressed as mM Trolox Equivalents (TE).

#### 2.3.5. Antimicrobial Activity

The antimicrobial activity of the hydrolates was investigated in accordance with a previously established procedure [21], with some minor modifications. Two target microorganisms, *Escherichia coli *(CECT 45) and *Listeria innocua *(CECT 910), were cultured in Mueller-Hinton broth (MHB) at 37 °C for 18 h. To determine the CFU, 180 µL of MHB containing 10^5^ CFU mL^−1^ diluted bacteria (1/1000 ratio in McFarland standard turbidity) and 10 µL of hydrolates solution were added per well in a 96-well plate. The microplates were incubated at 37 °C for 24 h under aerophilic conditions. The absorbance at 510 nm was measured at 24 h using a plate reader spectrophotometer (Thermo Scientific Electron APPLISKAN, Type: 2001, Waltham, MA, USA). Turbidity readings were used to determine the bacterial growth. The inhibitory effect was calculated using the following formula:

$$\%\mathrm{inhibition}=\frac{∆{\mathrm{Abs}}_{\mathrm{Reference}}-∆{\mathrm{Abs}}_{\mathrm{Assay}}}{∆{\mathrm{Abs}}_{\mathrm{Reference}}}\;\times \;100$$

where:

∆Abs_Reference_ is the increase in absorbance of the control sample.

∆Abs_Assay_ is the increase in absorbance of the test sample.

### 2.4. Marinated Meat Characterization

#### 2.4.1. Meat Proximate Composition

The moisture, total fat, and protein composition (%) as well as the pH were evaluated in five fillets. The moisture (%) was determined by drying the samples at 104 °C until to obtain constant weight, gravimetrically. Fat (%) was determined through extraction with chloroform/methanol (2:1) following Folch extraction method [22]. Protein (%) was determined using the Kjeldahl method [23].

#### 2.4.2. Microbiology of Marinated Meat

A 10 g portion of each sample was aseptically collected and homogenized with 90 mL of sterile peptone water using a laboratory blender (Stomacher R 400 Circulator) for 1 min. Serial decimal dilutions were then prepared in sterile peptone water, and 1 mL of each dilution was spread onto appropriate culture media. Mesophilic aerobic bacteria were enumerated using Plate Count Agar (Merck, 1.07881), after incubation at 30 °C for 72 h. Psychrophilic bacteria were counted on the same medium following incubation at 7 °C for 10 days. *Escherichia coli* and total coliforms were determined on Chromocult Agar (Merck, 1.10426) after incubation at 37 °C for 24–48 h. *Staphylococcus aureus* was identified on Baird Parker Agar (Merck, 1.05406) following incubation at 37 °C for 24–48 h. Results were expressed as Log_10_ CFU (colony forming units) per gram of sample. The detection limit for all methods was 10 CFU g^−1^, except for *S. aureus*, which had a detection limit of 100 CFU g^−1^.

#### 2.4.3. Instrumental Colour of Marinated Meat

The colour coordinates lightness (L), redness (a*, red/green axis), and yellowness (b*, yellow/blue axis) were analysed within the CIE Lab colour space, where CIE refers to the Commission Internationale de l’Éclairage (International Commission on Illumination). In addition, Hue angle, was calculated (h° = tan^−1^ (b*/a*)) as well as the saturation index or Chroma (C*) (C = (a* ^2^ + b* ^2^) ^0.5^). A total of four-colour measurements (carried out at room conditions of 21 ± 2 °C and 40–60% relative humidity) were taken for each fillet (two on each side), and the final value was determined as the mean of the readings.

#### 2.4.4. Oxidative Stability

Lipid oxidation was assessed by thiobarbituric acid reactive substances (TBA-RS) according to Sørensen & Jørgensen [24]. TBARS values were calculated from the standard (1,1,3,3-Tetraethoxypropane, TEP) curve and the results were expressed as mg of malondialdehyde per kg of sample (mg MDA kg^−1^). Protein oxidation was evaluated by measuring the carbonyl groups formed during incubation with 2,4-dinitrophenylhydrazine (DNPH) in 2 N HCl [25]. The absorbance measurement of protein concentration was detected at λ—280 nm using a spectrophotometer (evolution UV-Visible, Thermo Scientific, Waltham, MA, USA) with bovine serum (BSA) as standard. Protein oxidation was expressed as nmol carbonyls mg protein^−1^.

### 2.5. Statistical Analysis

Mean values and standard deviation were calculated. Three independent samples per batch were evaluated for the characterization of hydrolates and for the analysis of the effect of the hydrolates on meat marination. For the characterization of meat composition 5 independent samples were evaluated. One-way ANOVA was employed to find differences among treatments (hydrolates at different dosses) using the SPSS 21.0 statistical program (SPSS Inc., Chicago, IL, USA). If ANOVA detected significant differences between mean values, these were compared using Tukey’s test (*p* < 0.05). Another one-way ANOVA was carried out to evaluate the effect of storage on marinated samples. The relationship between the hydrophilic AA and the total phenolic content of the hydrolates was calculated using the Pearson correlation coefficient. Additionally, a two-way ANOVA was performed to evaluate the combined effects of hydrolate type and storage time on the parameters studied on meat.

## 3. Results and Discussion

### 3.1. Characterization of Hydrolates

The volatile composition of each hydrolate from *Lavandula luisiery*, *Thymus mastichina* and *Origanum virens* (mean percentage and standard deviations of each compound) was analyzed using gas chromatography–mass spectrometry methodology (GC-MS) (Table 1 and Table S1).

The main volatile compounds identified in *Lavandula luisiery* hydrolate were terpenoids and ketones like eucalyptol, *α*-cyclogeraniol, 2-cyclohexen-1-one, 3,4,4-trimethyl, alcanfor, 2-cyclopenten-1-one, 2,3,4,5-tetramethyl, 2-cyclopenten-1-one, 3,4,5,5-tetramethyl-, cis-verbenone. The rest of the isolated compounds showed less than 5% of the total compounds. *Thymus mastichina* hydrolate was rich in eucalyptol, α-terpineol, (-)-α-terpineol, L-4-terpineol. Similarly, the remaining compounds accounted for less than 5% of the total isolated compounds. Finally, the *Origanum virens* hydrolate was composed by thymol which accounted for almost 94% of the total volatile compounds isolated with carvacrol being the second major component.

The major volatile compounds found in the *L. luisiery* hydrolate were notably different from those typically dominant in *Lavandula* essential oil. The main characteristic of *Lavandula* spp. essential oil was its higher content in linalool/linalyl acetate and low camphor content [26]. However, in the hydrolate analyzed, the fundamental component is eucalyptol, with camphor in fourth position in terms of abundance. Notably, according to García-Custodio et al. [11], eucalyptol is also the second most abundant component in the essential oil of *L. luisieri*. The eucalyptol or 1,8-cineol is a bicyclic monoterpene found in the essential oils of various plants, including *Eucalyptus globulus*, *Rosmarinus officinalis*, *Origanum vulgare*, *Thymus vulgaris*, and *Coriandrum sativum* [27]. Monoterpenes, like eucalyptol or 1,8-cineol, demonstrate potent antibacterial activity against a broad spectrum of antibiotic-resistant pathogenic bacteria [28]. The main volatile compounds identified in the literature in the *Thymus* hydrolate were terpenoids (thymol, carvacrol, linalool, geraniol, *p*-cymene, *γ*-terpinene, limonene, *β*-caryophyllene), phenolic compounds (quinic acid, rosmarinic acid, caffeic acid, *p*-coumaric acid, syringic acid) and flavonoids (apigenin, luteolin, cirsimaritin) [29]. In contrast, in our study, the major terpenoids identified were eucalyptol and α-terpineol.

Changes in the composition of hydrolates fraction are common. According to Gavriil et al. [30], the absence of thymol and carvacrol in the hydrodistilled *Thymus* extracts may be attributed to the volatilization of these compounds into the essential oil fraction during the distillation process. The volatile profile of the *O. virens* hydrolate was similar to that usually described for *Origanum* essential oils, in which thymol is abundant but generally accompanied by higher proportions of carvacrol, p-cymene, or γ-terpinene [11]. However, the composition of *Origanum* essential oils can vary considerably depending on the species and chemotype [31]. The compositional differences between hydrolates and essential oils are generally notable, as has been previously reported; this is attributed to the distillation process, which favors the partition of highly volatile oxygenated monoterpenes into the aqueous fraction, whereas hydrocarbons and less polar compounds tend to concentrate in the essential oil [30]. Inouye et al. [6] compared the volatile composition of 44 hydrolates with their corresponding essential oils and concluded that in 42% of the cases the compositions differed, suggesting that the bioactivity of hydrolates is not always equivalent to that of the essential oils. Several unique components were found to be rich in hydrolates but not so in the essential oils. In this line, other study of the composition of essential oils and their corresponding hydrolates obtained from six plants from *Lamiaceae* family (*Thymus vulgaris* L., *Thymus pannonicus * All,. *Lavandula angustifolia* L., *Lavandula × intermedia*, *Origanum vulgare* L., and * Origanum vulgare* var. *aureum* L.) by GC-MS revealed different compositions of bioactive compounds [26]. They reported that average chemical composition of essential oils typically includes two major classes, hydrocarbon compounds (terpenes) and oxygenated compounds (terpenoids). Hydrocarbon terpenes are nonpolar and exhibit low water solubility, which explains their predominant presence in the essential oil fraction. In contrast, hydrolates are rich in many oxygenated compounds which are more water-soluble. Among the most common oxygenated monoterpenes found in hydrolates were borneol, carvacrol, linalool, 1,8-cineole, camphor, terpinen-4-ol, and thymol [7]. Hydrolates typically contain less than 1 g L^−1^ (0.10%) of water-soluble volatile organic compounds derived from the essential oils, which remain dissolved in the aqueous phase [32], so that important differences between the essential oil and the hydrolate are expected.

The pH, microbiological counts of the hydrolates, the phenolic compounds content and the bioactive properties of the three hydrolates studied—*L. luisiery*, *T. mastichina*, and *O. virens*—were evaluated and presented in Table 2.

The pH of the studied hydrolates was measured, resulting in values below 4.3, which agrees with the acidic pH reported in the literature [33]. The microbial composition of the hydrolates was analysed in order to test their safety and suitability for meat marination. All hydrolates showed undetectable microbial counts (<1 log CFU mL^−1^) of aerobic mesophilic, molds and yeasts, *Clostridium* spp., and *Bacillus* spp. These results confirm the initial microbial stability of the extracts and support their use as safe natural ingredients for food applications. Hydrolates require careful handling to maintain their stability over time. In this regard, it is important to note that they should be stored in aseptic containers that are completely filled to eliminate any air gap, sealed and kept in a cool environment. Garneau et al. [34], in their study on hydrolate storage, reported that the major constituents of hydrolates remained stable for at least 12 months at room temperature. The low pH (3.9–4.3) and the presence of antimicrobial compounds on the hydrolates enhances their stability during the storage. Additionally, there is a risk of contamination during packaging if proper hygienic practices are not followed.

The shelf-life of hydrolates largely depends on their chemical composition and the intrinsic antimicrobial activity of the compounds they contain. *O. virens* hydrolate exhibited both the highest phenolic concentration (272.7 ± 22.0 mg GAE L^−1^) and the greatest hydrophilic antioxidant activity values (6.3 ± 0.1 µmol mL^−1^ Trolox equivalents), significantly (*p* < 0.001) surpassing *L. luisiery* and *T. mastichina*, which showed low phenolic content and corresponding antioxidant activity. This result is consistent with the results obtained from the Pearson correlation analysis, which revealed a strong and statistically significant positive correlation between phenolic content and antioxidant activity (r = +0.983, *p* < 0.01). These findings support the potential use of *O. virens* hydrolate as a natural antioxidant agent in food preservation, contributing to product stability and shelf-life extension. Among the many properties that are often attributed to plant essential oils, the antioxidant property certainly stands out, Saleh et al. [35] indicating that phenolic compounds, which can act as free radical scavengers, are the main contributors to the antioxidant capacity of these extracts [36].

The antimicrobial activity of the hydrolates against *Escherichia coli* and *Listeria innocua* was evaluated by measuring their percentage of inhibition in vitro. Overall, the inhibition percentages against *L. innocua* were higher (ranging from 19.2% to 25.4%) compared to those observed against *E. coli* (4.1% to 5.1%). This trend is consistent with the known differences in cell wall structure between these two bacteria. Generally, Gram-negative bacteria are more resistant to essential oils than Gram-positive bacteria [37]. The cell wall of Gram-negative bacteria is more resistant to the activity of essential oils and their components, primarily because it restricts the penetration of hydrophobic molecules more effectively than the Gram-positive cell wall [38].

For instance, *E. coli*, a Gram-negative bacterium, possesses an outer membrane enriched with lipopolysaccharides, which confers enhanced resistance to a wide range of antimicrobial agents, including plant-derived compounds. In contrast, *L. innocua*, a Gram-positive bacterium, lacks this outer membrane, rendering it generally more susceptible to such treatments. The antimicrobial activity was similar in all hydrolates (*p* ≥ 0.05), although slightly higher in *Origanum virens*. Pearson correlation coefficient between total phenolic content and *L. innocua* percentage inhibition was calculated, revealing a strong and significant positive correlation (r = +0.739; *p* < 0.01), which indicates that differences in the levels of phenolic compounds content would also be related to the antimicrobial activity.

Based on the results and the observed differences in total phenolic content, antioxidant activity appears to be closely linked to phenolic compounds. However, the antimicrobial activity may also be influenced by other constituents, as no significant differences were observed among the hydrolates despite variations in their phenolic content. While the antibacterial effects of essential oil extracts are often attributed to their phenolic fractions such as thymol and carvacrol found in *O. virens* hydrolate [39]—other non-phenolic compounds may also contribute. For example, eucalyptol and α-terpineol, present in *L. luisiery* and *T. mastichina* species, are known to exhibit antibacterial properties. Moreover, these components can act synergistically, enhancing the overall antimicrobial effectiveness [40].

Due to their antimicrobial properties, hydrolates are considered suitable candidates for use as natural antimicrobials in the food industry and as sanitizing agents for equipment, machinery, and work surface [32]. Due to their aqueous nature, hydrolates can be easily rinsed from surfaces and do not impart a strong or persistent odor, unlike essential oils. Furthermore, hydrolates may offer a promising alternative for the prevention of biofilm formation on processing equipment and work surfaces [32]. The microbiological safety and moderate antimicrobial activity of all tested hydrolates further support their use as functional, natural ingredients in sustainable food preservation strategies. In addition, the superior antioxidant capacity and phenolic content of *Origanum virens* hydrolate suggest its potential as a natural antioxidant and preservative in food systems, contributing to shelf-life extension and improving product quality.

### 3.2. Application of Hydrolates for Meat Marination

#### 3.2.1. Effect on Microbial Counts of Meat

The microbial counts of fillets were evaluated after the marination with hydrolates at different doses for 24 h (1 day) and after 8 days of storage (9 days) (Table 3). The effects of both the hydrolate type (*Lavandula luisiery*, *Thymus mastichina* and *Origanum virens*) and the applied dose were analysed. Meat is highly susceptible to biochemical and microbial deterioration, particularly during preservation, mainly due to its high a_w_ [41].

At day 1, a dose-dependent effect (*p*-Dose < 0.05) on mesophilic counts (log CFU g^−1^) was observed exclusively for *L. luisiery* hydrolate, with the highest proportion (1:2) leading to a significant reduction in mesophilic levels. *T. mastichina* and *O. virens* hydrolates did not show this effect. Regarding the global effects of hydrolates, the highest counts of mesophilic were found at *T. mastichina* 1:1.5 and the lowest at *L. lusiery* 1:2, the rest showed intermediate levels. After the refrigerated storage, mesophilic counts were significantly increased in all groups and at day 9, they followed a similar pattern as at day 1, the highest counts of mesophilic were found at *T. mastichina* 1:1.5 and the lowest at *L. luisiery* 1:2.

At day 1, psychrophilic counts were not affected by the dose of hydrolate. The highest levels were found in control while the lowest levels were found at the highest dose of *L. luisiery* and *O. virens* (1:1). After 9 days of storage, microbial counts increased significantly in all groups. In addition, at day 9 psychrophilic counts were affected by the dose of *T. mastichina* although the lowest counts were found at dose 1:1. Regarding individual differences, the highest counts were found in *T. mastichina* 1:1.5 while the lowest levels were found in *O. virens* (1:1).

Total coliforms counts were not affected by the hydrolated dose. On day 1, the highest levels were found in control while the lowest levels were found in *O. virens* (1:2). After the storage, the counts were not modified except for *L. luisiery* 1:1, which were significantly reduced. At the end of storge, the counts were similar in all groups. *E. coli* counts were below the detection limit of the method (<1 log CFU g^−1^) or close to the limit in all groups. Counts did not show significant differences due to the application of marinates at day 1 or day 9. The counts during storage remained unchanged in all groups and also in control. All hydrolates exhibited similar and low antimicrobial activity against *E. coli* (Table 2) in the in vitro analysis. Consistent with these results, only slight reductions in total coliforms were observed in pork fillets on day 1. Comparable reductions in total coliform and *E. coli* counts were observed on day 9 for total coliforms and on both day 1 and day 9 for *E. coli* (Table 3).

*S. aureus* was dose dependent for *L. luisiery* hydrolate. On day 1, the levels of *S. aureus* were reduced at doses of 1:1.5 and 1:2 compared to 1:1. Surprisingly for *O. virens* the highest doses increased the levels of *S. aureus* therefore, an increase in hydrolate dosage does not necessarily correlate with enhanced antimicrobial efficacy [42]. This is consistent with the findings of Di Vito et al. [43], who reported that increasing the concentration of hydrolates does not necessarily result in a linear enhancement of their bioactivity. On day 1, *S. aureus* counts were highest in the *L. luisiery* 1:1 treatment compared to the control and other hydrolates, indicating that *L. luisiery* 1:1 at low doses promoted microbial growth of *S. aureus*. The other hydrolates did not significantly affect *S. aureus* levels at this time. During storage, a reduction in *S. aureus* counts was observed in some groups, including the Control, *L. luisiery* 1:1, *T. mastichina* 1:2, and *O. virens* 1:1.5 and 1:2, whereas in the remaining groups, bacterial counts remained stable. By day 9, *S. aureus* levels were similar across all treatments, with no observable dose-dependent effect.

In general, hydrolates seemed to exhibit only limited antimicrobial effect during meat marination, with *L. luisiery* and *O. virens* being the most effective. In the case of *L. luisiery* hydrolate, eucalyptol (also known as 1,8-cineole) has been identified as one of its principal volatile constituents and is well recognized for its antimicrobial properties. Several studies have reported that essential oils rich in eucalyptol demonstrate moderate to strong antibacterial activity against a wide range of microorganisms [44]. Although the precise mechanism of action of this compound remains unclear, it has been suggested that eucalyptol targets already compromised bacterial membranes, leading to the inactivation of essential cellular components [45]. Notably, eucalyptol derived from *Lavandula officinalis* essential oil has shown greater antibacterial activity than gentamicin [46]. In our analysis, camphor was identified as the fourth most abundant compound in the hydrolate, accounting for 11.03% of the total composition, and may also contribute to its antibacterial potential, as previously reported against Gram-negative bacterial strains [46,47]. Therefore, the antimicrobial effect observed during meat marination with *L. luisiery* could be partly attributed to a possible synergistic interaction between eucalyptol and camphor. On the other hand, *O. virens* hydrolate is particularly rich in thymol and carvacrol, which is consistent with the findings of Kachur and Suntres [48], who explored various mechanisms underlying the antimicrobial activity of these compounds, confirming their role in disrupting bacterial membranes and inhibiting the function of efflux pumps, bacterial motility, and membrane-associated ATPases. These results are consistent with the study conducted by Gavrill et al. [30] who found that *origanum* plant aqueous extracts obtained as by-products of essential oil purification exhibited prominent bactericidal properties against the *Salmonella typhimurium* FS8 strain inoculated on marinated pork meat stored at 4 °C. Differences with literature about the antimicrobial effect of hydrolates might be related to the variable composition of hydrolates and to the lower levels of bioactive compounds compared to the essential oil or pure compounds.

Although numerous in vitro studies have demonstrated the antimicrobial activity of plant extracts, relatively few have investigated their effectiveness in food systems. This discrepancy may be attributed to the reduced antimicrobial activity of plant extracts in complex food matrices compared to that of purified bioactive compounds. It is widely recognized that the effectiveness of plant extracts as antimicrobials can be significantly reduced when applied in food matrices Such diminished efficacy is often linked to the use of crude extracts, which commonly contain compounds in glycosidic forms. The presence of sugar moieties in these forms might reduce their antimicrobial potential or, in some cases, even support microbial growth [49]. This could partly explain the higher levels of *S. aureus* of *Lavandula luisiery* (1:1) marinated meat.

Pork marination with hydrolates slightly limited microbial growth. Foods with complex compositions, such as meat, pose challenges for the effective use of natural antimicrobials due to their intrinsic properties, including heterogeneous structure, neutral pH, and high levels of lipids and proteins [50]. In fact, within such complex systems, lipids and proteins can strongly bind to bioactive phenolic compounds, thereby diminishing their antimicrobial potency [30]. This would explain why in vitro studies are generally more successful than studies in situ in meat matrices.

#### 3.2.2. Effect on the Instrumental Colour of Meat

Instrumental colour was evaluated in the marinated pork fillets at day 1 and day 9 (Table 4). The effect of the hydrolate dose was evaluated as well as the individual effects of the 3 types of hydrolates studied. CIE L* (lightness) values were not affected by the hydrolate dose used during marination, either on day 1 or day 9. However, marination itself slightly increased the lightness of the meat compared to the control (although differences were only significant on day 9). Storage did not significantly alter the initial lightness values (*p* ≥ 0.05). Changes such as increased paleness or discoloration can negatively affect consumer perception, since colour is widely regarded as an indicator of both quality and freshness and often represents the primary limiting factor in meat shelf life [51]. These changes could be explained by the presence of exudate or surface moisture on meat products, which may increase the reflection of incident light. In this context, the marination process could contribute to this effect, as marinated products typically retain a greater amount of free or loosely bound surface water, along with dissolved compounds such as salts, sugars, and soluble proteins. On the other hand, the increased paleness of meat could also be attributed to the acidic pH of the hydrolates which could promote a partial denaturation of proteins which may modify the appearance of the meat surface.

The redness (CIE a*) of meat remained unaffected by the marination process (*p* > 0.05), and similar values were found in all groups at day 1 and after 9 days of storage. This would indicate that redness values was well preserved during the storage. However, mean values of redness tended to decrease after the marination at day 1, although differences were not significant. The characteristic red colour of meat is primarily attributed to myoglobin, a heme protein located in skeletal muscle that is responsible for oxygen transport within muscle fibers [52]. The colour of meat depends largely on the amount of myoglobin present and its chemical state. When freshly cut meat is exposed to air, myoglobin binds with oxygen to form oxymyoglobin, giving the meat a bright red colour that is often associated with freshness. Colour stability during storage may also be explained by the protective effect of vacuum packaging, which limits oxygen exposure and thereby slows the oxidative processes that would otherwise lead to colour deterioration over time.

The CIE b* parameter (yellowness) exhibited a similar trend to CIE a* and remained unaffected by the marination process, as well as by the type or dosage of hydrolate. In addition, after the refrigerated storage (day 9), marinated fillets presented similar values as control. However, significant increases in yellowness were observed in all groups (including the control group) during the storage period, except the fillets marinated with *L. lusiery* 1:1 and *O. virens* 1:2. The increase in meat yellowness during refrigerated storage is associated with enhanced lipid oxidation over time [53]. According to the results, the application of some marination conditions could reduce the yellowness increase during storage, preserving the original meat colour. This could indicate that the antioxidant effect of some of the hydrolates could reduce the lipid oxidation development of meat during the storage.

Chroma was not affected by the hydrolate dose for the marination. In addition, control and marinated meat presented similar values. This effect was maintained on day 9. The lack of effect of marination on CIE a* and b* could explain these results since Chroma is calculated with both parameters. During the storage, values of Chroma remained constant in control, *L. luisiery* (1:1), *T. mastichina* (1:1.5 and 1:2), *O. virens* (1:2), while this parameter increased significantly in the other groups. On the other hand, Hue angle did not show significant differences either on day 1 or day 9 and remained unaffected during storage. Changes of Chroma and Hue are caused by the variations of CIE a* and b* since both are calculated using these parameters. The lack of effects of marination on the parameters Chroma and Hue indicate that only slight colour changes in lightness are produced in the fillets by the marination process.

#### 3.2.3. Effect on the Oxidative Stability of Meat

The oxidative status of pork fillets was evaluated after marination with hydrolates at different doses (Table 5). The effect of the dose was analyzed as well as the individual contribution of the three hydrolates studied (*Lavandula luisiery*, *Thymus mastichina*, and *Origanum virens*). Oxidation was assessed by measuring the lipid oxidation development, evaluated by the level malondialdehyde (MDA, mg kg^−1^) and the protein oxidation development analyzed by the carbonyl levels (nmol mg of protein^−1^).

On day 1, lipid oxidation levels remained very low in all groups, which could be attributed to the vacuum packaging as well as the low fat content of the meat. The origanum-treated samples showed the lowest levels (0.04 mg MDA kg^−1^), while the highest values were found in the lavender-treated fillets, particularly at 1:1.5 (0.11 mg MDA kg^−1^). *Thyme* and *origanum* treatments maintained similar oxidative levels to the control at all tested doses. No significant dose-dependent effects were observed for any of the studied hydrolates. After 9 days of refrigerated storage, lipid oxidation values were similar to those recorded on day 1 in all samples although mean values of control were slightly increased. In lavender-treated samples, no significant differences were observed among doses, although all showed higher MDA than the control. Dose-dependent effects were only observed in the thyme-group, where the 1:1.5 and 1:2 dose resulted in lower MDA levels compared to 1:1, indicating a potential antioxidant action at higher doses. The lowest lipid oxidation levels were observed in *O. virens* 1:1.5, while highest values were found in lavender-treated samples.

These results suggest that *T. mastichina* and especially *O. virens* hydrolates may provide better oxidative protection during storage, particularly at specific dilutions. This is consistent with the phenolic content and the in vitro antioxidant activity. *O. virens* exhibited strong antioxidant activity and a high phenolic content (Table 2), thus proving effective even at low doses. In contrast, *T. mastichina* showed intermediate to low values and required higher doses of hydrolates to demonstrate effectiveness. Moreover, the decrease in lipid oxidation corresponds with the reduced yellowness development (CIE b*, Table 4) detected in meat marinated with *T. mastichina* and *O. virens* during refrigerated storage. In general, increases in meat yellowness are associated with the development of lipid oxidation.

Oxidative reactions occur during the conversion of muscle into meat, meat processing, and storage, and constitute one of the main causes associated with the degradation of meat quality [41]. Phenolic compounds have the ability to inhibit oxidation reactions by slowing down lipid oxidation, binding to proteins, and forming complexes with them [54]. In this study, essential oil from *Origanum* has shown promising effects in delaying lipid oxidation. The antioxidant effect of *Origanum* could be largely attributed to the high polyphenol content of its essential oil. In addition, major compounds such as carvacrol, thymol, p-cymene, and γ-terpinene are primarily responsible for its antioxidant activity [55]. Thymol and carvacrol are the main and second most abundant components of the *O. virens* hydrolate used in our study, which could explain its antioxidant activity, although less pronounced than that of the essential oil.

In contrast, *L. luisiery*, on the other hand, demonstrated limited antioxidative potential and even a possible pro-oxidative tendency (Table 5). This behavior may be related to the oxidation of the hydrolate’s lipophilic fraction or to the presence of pro-oxidant constituents such as salts, metals, or acidic compounds. The higher lipid oxidation observed in samples treated with *L. luiseiri* compared with the control may also stem from the lipidic components present in the hydrolate—small droplets of essential oils that are highly susceptible to oxidation—which could oxidize during marination and, in turn, promote oxidative reactions in the meat, as reflected in the TBA values.

Protein oxidation values, measured as carbonyl formation (Table 5) did not show significant variation between doses on day 1. The highest carbonyl levels were observed in *L. luisiery* 1:1.5, while the lowest were found in *T. mastichina* 1:2, the control presented intermediate values. After 9 days of refrigerated storage, the values remained stable among all treatments, except for *O. virens* 1:1, which exhibited a noticeable increase.

Protein oxidation in meat starts from *post-mortem* and continues during refrigerated/frozen storage and processing and causes structural modifications, nutritional losses and physicochemical changes, which affect the quality and sensory characteristics of meat products [56]. Protein oxidation has been demonstrated to impact the quality of muscle foods in various aspects [54]. It promotes the formation of volatile compounds which contribute to off-flavors and off-odors, negatively influencing consumer acceptance. Structural changes, including protein cross-linking and aggregation reduce water-holding capacity and tenderness [57]. Nutritionally, oxidation may decrease protein digestibility and lower the bioavailability of essential amino acids, depending on the extent of oxidation [58].

Protein oxidation also plays a key role in promoting lipid oxidation, contributing to rancidity, texture degradation, and reduced storage stability [59]. Although protein and lipid oxidation are closely interrelated phenomena in meat systems, protein oxidation has historically been understudied and remains less well understood. In this respect, *L. luisiery* samples presented the highest protein and lipid oxidation extent at day 1, which could be explained by the relationship between both reactions. However, on day 9, lipid oxidation changes did not show a similar pattern as protein oxidation development. This disparity may be attributed to several factors, including the complex chemistry involved in protein oxidation, the limited availability of specific analytical methods to evaluate it in food matrices. In general, the antioxidant effect of hydrolates seems to depend more on the type of hydrolate and dose used than on storage time alone. In this context, other authors have reported that the antioxidant activity of hydrolates is significantly lower than that of the corresponding essential oils [60,61]. Despite their lower phenolic content, hydrolates remain promising as natural antioxidants due to their recognized safety, subtle sensory profile, and good compatibility with food matrices.

A clear dose-dependent effect was observed at the microbiological level, particularly for the *L. luisiery* hydrolate, which showed a significant antimicrobial activity at the highest concentration (1:2), resulting in reduced counts of mesophilic compared to the lower doses. However, this dose-dependent effect was not evident for the oxidative parameters, where values remained low or exhibited inconsistent variation across treatments. This absence of a clear dose-dependent antioxidant effect may be attributed to the overall low oxidation levels observed in all groups, especially at the beginning of storage, which may have limited the ability to detect significant differences between doses. It is likely that oxidative stress was not sufficiently elevated to reveal a potential protective effect of the hydrolates, thereby preventing the observation of a clear dose–response relationship in terms of antioxidant activity. Additionally, because hydrolates are rich in hydrophilic compounds, their antioxidants may have limited efficacy in inhibiting lipid oxidation within complex products such as meat.

#### 3.2.4. Global Effects of Hydrolates and Storage on Meat

The general effects of the hydrolates and the time of storage were evaluated by a two-ways ANOVA (Table 6). Most microbiological counts were influenced by the effects of hydrolate and storage except for *E. coli*, which was below the detection limit. This agrees with the slight antimicrobial effect showed in the marinates with *L. luisiery* and *O. virens* (Table 3). In addition, storage had also a significant effect on all microbial counts except for *E. coli* (Table 6). Concretely, mesophilic and psychrophilic counts increased, while coliforms and *S. aureus* decreased or remained unchanged after 9 days of refrigeration. *E. coli* did not show changes, and the effect was not significant (Table 3). A significant interaction between both factors of the two-ways ANOVA (hydrolate x storage) was also observed in *S aureus*, which agrees with the increase in this microbial group in some types of hydrolate, like *L. luisiery* at day 1, while at day 9 the levels remained similar as the other types of hydrolate.

All instrumental colour parameters were affected by the application of hydrolates, while storage time influenced CIE a*, CIE b*, and Chroma (Table 6). Hydrolate treatment generally increased lightness and reduced both redness and yellowness, whereas storage tended to increase redness or yellowness (Table 4). Notably, these effects were not detected by the one-way ANOVA, but were apparent when using two-way ANOVA. Given this and considering the importance of meat colour in consumer choice, further sensory evaluation should be required to determine whether the marination process may influence purchasing decisions. In this regard, sensory evaluation should also be needed to determine whether the aromas imparted by the hydrolate are acceptable to consumers. Sensory analysis from previous studies showed no negative effects in the odour and appearance when atomized thyme and lavender hydrolates were applied to trout fillets, while microbial growth was reduced during refrigerated storage [62].

In the two ways-ANOVA, lipid oxidation (TBA-RS values) was affected by hydrolate, whereas protein oxidation (carbonyl formation) was not, despite the slight increases at some conditions (and at certain dilutions of *L. luiseiri* and *O. virens* increased carbonyl formation) in the one-way ANOVA. Storage did not affect both oxidative parameters (Table 6). Lipid oxidation agrees with the changes shown in Table 5 for the differences in the TBA-RS values among hydrolates and the greater antioxidant effect shown by *O. virens* and *T. mastichina* compared with *L. luisieri*.

Some quality attributes of the marinated meat, such as microbial growth and colour characteristics could be influenced by the acidic pH of the hydrolates. In fact, these parameters were slightly altered by the marination process. On the other hand, protein oxidation, assessed through carbonyl formation, was not affected; however, additional methods would be required to more thoroughly evaluate protein denaturation. Other parameters, including water-holding capacity and texture of marinated meat, should also be examined in future studies. The pH of hydrolates can induce protein denaturation, mainly by shifting the environment toward the isoelectric point, although the severity may depend on hydrolate pH, application method, contact time, and the food’s buffering ability.

Although the antimicrobial activity of plant extracts has been extensively demonstrated in vitro, there is a relative lack of studies evaluating their efficacy in food systems. This is probably because plant extracts are less effective when applied to complex food matrices. To date, only one recent study has specifically assessed the use of hydrolates in meat products. Gavriil et al. [30] investigated the in vitro antimicrobial activity of nine hydrodistilled aqueous plant extracts (hydrolates) including basil, calendula, centrifuged origanum, corn silk, laurel, origanum, rosemary, spearmint, and thyme—against three strains of *Salmonella asmurium* as well as in pork meat. Their findings showed that marination was a rapid and effective intervention. However, consistent with the results observed in the current study, the antimicrobial effect was more pronounced in vitro than in vivo. To improve their efficacy in food systems, the study also tested the combination of hydrolates with essential oils, which significantly enhanced their antimicrobial activity. This may also contribute to standardizing the composition of hydrolates, whose characteristics vary considerably according to the distillation conditions applied during their production. In this context, D’Amato et al. [63] also demonstrated a synergistic effect by combining hydrolates with essential oils (*Origanum vulgare* var. *hirtum* and *Coridothymus capitatus*) against *Listeria monocytogenes*. Their findings suggest that the combined use of both products can open new avenues for applications by enhancing antimicrobial efficacy while reducing the required concentrations.

## 4. Conclusions

Hydrolates from *Lavandula luiseiri*, *Thymus mastichina*, and *Origanum virens* were microbiologically safe and displayed notable antioxidant potential, although their antimicrobial activity was only slight. *O. virens* hydrolate had the highest phenolic content and strongest antioxidant activity, highlighting its potential as a natural preservative to improve oxidative stability in meat, while all hydrolates showed similar antimicrobial effects. Pork marination with hydrolates limited lipid oxidation, although the reduction in microbial growth was only marginal, with both effects varying according to plant source and dilution. *O. virens* and *T. mastichina* hydrolates provided greater protection against lipid oxidation than *L. luisiery*, with *O. virens* at higher concentrations showing the strongest antioxidant effect during storage. However, marination process should be optimized since it induced slight colour changes. Despite being less concentrated than essential oils, hydrolates retain bioactive polyphenols that confer beneficial effects. Their use alone may be insufficient for long-term preservation of fresh meat. However, combining them with essential oils, partially incorporating them into processed products such as frankfurters, or pairing them with synergistic strategies like high-pressure processing or modified-atmosphere packaging could enhance their effectiveness.

Several limitations should be addressed in future research. The lack of sensory evaluation—particularly relevant given the strong aromatic profiles of hydrolates and the potential colour changes during marination—prevents a meaningful assessment of their technological feasibility in real meat systems. Future works should also prioritize other aspects such as proteomics, lipidomics, or modelling of oxidation kinetics, to achieve a more comprehensive understanding of the mechanisms and practical potential of hydrolates as natural preservatives.

## Figures and Tables

**Table 1:** Volatile organic compounds identified in hydrolates of *Lavandula luisiery*, *Thymus mastichina*, and *Origanum virens*.

*Lavandula luisiery*					*Thymus mastichina*					*Origanum virens*				
Compound	Retention Index (RI)	Mean ± SD			Compound	Retention Index (RI)	Mean ± SD			Compound	Retention Index (RI)	Mean ± SD		
Ethane, 1,1-dimethoxy-	608	207	±	4	cis-3-Hexen-1-ol (*)	855	314	±	185	Ethane, 1,1-dimethoxy-	608	116	±	15
3-Methyl-1-buten-3-ol	614	529	±	13	Benzaldehyde (*)	959	393	±	8	Butanal, 3-methyl-	647	60	±	2
2-Butanone, 3-methyl-	643	276	±	21	1-Octen-3-ol	982	154	±	6	Methyl 2-methylbutanoate	760	35	±	3
2-Butenal, 3-methyl	766	356	±	9	Eucalyptol	1031	119,974	±	2170	Furfural	828	59	±	4
Furfural	828	329	±	2	β-Linalool (*)	1105	6409	±	260	trans-Hex-2-enal (*)	852	34	±	3
cis-3-Hexen-1-ol (*)	855	696	±	27	(1R)-(+)-Norinone	1140	739	±	57	cis-3-Hexen-1-ol (*)	855	207	±	8
Benzaldehyde (*)	959	1487	±	63	trans-(-)-Pinocarveol	1144	882	±	21	trans-2-Hexenol	872	114	±	10
1-Octen-3-ol	982	809	±	13	(-)-Camphor	1147	259	±	3	Benzaldehyde (*)	959	78	±	6
Eucalyptol	1031	28,580	±	244	Pinocarvone	1166	267	±	3	1-Octen-3-ol	982	866	±	14
2-Cyclohexen-1-one, 3,4,4-trimethyl-	1082	13,622	±	24	(-)-α-Terpineol	1183	17,395	±	489	1,4-Cineol	1013	23	±	0
2-Cyclopenten-1-one, 3,4,5,5-tetramethyl-	1094	7225	±	24	L-4-terpineol	1189	11,630	±	454	m-Cymene	1023	10	±	1
2-Cyclopenten-1-one, 2,3,4,5-tetramethyl	1116	7414	±	117	α-Terpineol (*)	1212	51,689	±	903	Eucalyptol	1031	67	±	10
α-Isophorone	1122	1179	±	27	Verbenone	1223	390	±	11	trans-(-)-Pinocarveol	1144	2490	±	73
(1R)-(+)-Norinone	1140	2757	±	34	cis-Carveol	1229	280	±	5	Borneol	1177	1204	±	19
trans-(-)-Pinocarveol	1144	1992	±	16	cis-Geraniol (*)	1262	515	±	20	4-Terpineol	1205	354	±	21
Camphor	1149	12,134	±	22	Cumyl alcohol	1298	133	±	21	α-Terpineol (*)	1212	277	±	152
Pinocarvone	1166	1300	±	17	Elemol	1565	150	±	8	p-Cymen-8-ol	1215	14	±	1
α- Cyclogeraniol	1188	16,101	±	271	Spathulenol	1596	10	±	0	Thymol	1323	132,789	±	1914
(-)-Myrtenol	1204	1183	±	11	γ-Eudesmol	1651	25	±	1	Carvacrol (*)	1326	3061	±	42
cis-Verbenone	1216	5288	±	152	β-Eudesmol	1671	37	±	2	Thymol acetate	1367	49	±	16

(*) Volatile compounds were identified by the analysis of commercial standard. Mean peak areas (±SD, Standard deviation) Results are expressed as arbitrary area units (AAU) × 10^5^.

**Table 2:** Microbiological analysis of hydrolates, phenolic compounds content and their antimicrobial and antioxidant bioactivity.

	*Lavandula luisiery*			*Thymus mastichina*			*Origanum virens*			*p*-Value
**pH**	3.90 c	±	0.03	4.29 a	±	0.4	4.12 b	±	0.04	***
**Microbiological Counts (log CFU g^−1^)**										
Mesophilic	<1			<1			<1			ns
Molds and yeasts	<1			<1			<1			ns
*Clostridium*	<1			<1			<1			ns
*Bacillus*	<1			<1			<1			ns
**Phenolics compounds content ^1^**	9.4 b	±	0.2	4.2 b	±	0.1	272.7 a	±	22.0	***
***BIOACTIVITY***										
***Antioxidant activity***										
Hydrophilic AA ^2^	0.1 b	±	0.0	0.1 b	±	0.0	6.3 a	±	0.1	***
Lipophilic AA ^2^	ND			ND			2.5	±	0.4	
**Antimicrobial activity (% Inhibition)**										
*E. coli*	4.1	±	6.1	4.7	±	6.7	5.1	±	6.8	ns
*L. innocua*	19.2	±	14.4	22.7	±	14.6	25.4	±	19.4	ns

Different lowercase letters (a, b, c) in the same row indicate statistically significant differences between hydrolates. Significance levels: ns = not significant, *** = *p* < 0.001; ^1^ (mg GAE L^−1^) ^2^ (Trolox equiv., mM); ND = Not Detected.

**Table 3:** Microbial counts (log CFU g^−1^) of fillets marinated with different doses of hydrolates (1:1, 1:1.5, and 1:2) of *Lavandula luisiery*, *Thymus mastichina*, and *Origanum virens* during 1 and 9 days of storage.

Storage	Control			*Lavandula luisiery*										*Thymus mastichina*													*Origanum virens*							*p*-Value
(Days)				1:1			1:1.5			1:2			*p*-Dose	1:1			1:1.5			1:2			*p*-Dose	1:1			1:1.5			1:2			*p*-Dose	
Mesophilic																																		
1	4.8 AB	±	0.5	4.1 aBC	±	0.1	4.2 aBC	±	0.2	3.7 bC	±	0.2	*	4.5 ABC	±	0.5	5.1 A	±	0.1	4.5 ABC	±	0.5	ns	4.1 BC	±	0.2	4.2 ABC	±	0.1	4.3 ABC	±	0.1	ns	***
9	6.0 ABC	±	0.1	5.9 ABCD	±	0.2	5.4 CD	±	0.4	5.3 D	±	0.4	*ns*	6.2 bAB	±	0.1	6.5 aA	±	0.2	6.3 abAB	±	0.1	*	5.8 BCD	±	0.1	5.4 CD	±	0.2	5.3 CD	±	0.5	ns	***
*p-storage*	*			***			*			*				**			***			**				***			***			*				
Psychrophilic																																		
1	4.1 A	±	0.4	3.5 ABC	±	0.3	3.2 ABC	±	0.5	3.0 C	±	0.1	*ns*	3.8 ABC	±	0.5	4.1 AB	±	0.6	3.4 ABC	±	0.4	ns	3.0 C	±	0.1	3.1 BC	±	0.3	3.2 ABC	±	0.2	ns	**
9	6.0 BCD	±	0.1	5.7 CDE	±	0.2	5.5 DE	±	0.4	5.5 DE	±	0.2	*ns*	6.1 bABC	±	0.1	6.5 aA	±	0.2	6.4 aAB	±	0.1	**	5.9 CD	±	0.2	5.5 DE	±	0.2	5.3 E	±	0.3	ns	***
*p-storage*	**			***			**			***				**			**			***				***			***			***				
Total coliforms																																		
1	2.1 A	±	0.7	1.7 AB	±	0.3	1.5 AB	±	0.3	1.4 AB	±	0.1	*ns*	1.9 AB	±	0.7	2.2 A	±	0.4	1.8 AB	±	0.4	ns	1.0 AB	±	0.1	1.0 AB	±	0.1	0.9 B	±	0	ns	**
9	1.3	±	0.3	1.0	±	0.1	1.5	±	0.5	1.0	±	0.2	*ns*	1.3	±	0.6	1.8	±	0.7	1.5	±	0.6	ns	1.2	±	0.4	0.9	±	0.0	1.0	±	0.1	ns	ns
*p-storage*	ns			*			ns			ns				ns			ns			ns				ns			ns			ns				
*E. coli*																																		
1	<1			<1			<1			1.1	±	0.2	*ns*	0.9	±	0.1	1.1	±	0.2	0.9	±	0.1	ns	0.9	±	0.1	<1			<1			ns	ns
9	<1			<1			0.9	±	0.1	<1			*ns*	<1			<1			0.9	±	0.1	ns	*<1*			<1			<1			ns	ns
*p-storage*	ns			ns			ns			ns				ns			ns			ns				*ns*			ns			ns				
*S. aureus*																																		
1	2.7 B	±	0.5	3.5 aA	±	0.3	2.3 bB	±	0.3	2.3 bB	±	0.2	**	2.2 B	±	0.3	2.1 B	±	0.3	2.3 B	±	0.2	ns	2.0 B	±	0.1	2.6 B	±	0.2	2.6 B	±	0.2	*	***
9	<2			2.2	±	0.5	2	±	0.1	2.1	±	0.2	*ns*	<2			2.3	±	0.3	<2			ns	2	±	0.1	2.0	±	0.1	2.0	±	0.1	ns	ns
*p-storage*	***			*			ns			ns				ns			ns			*				ns			**			*				

Different lowercase letters in the same row indicate significant differences between doses for each hydrolate (*p*-Dose). Different uppercase letters in the same row indicate significant differences between groups (*p*-value). Significance levels: ns = not significant, * = *p* < 0.05, ** = *p* < 0.01, *** = *p* < 0.001.

**Table 4:** Instrumental color measurement of fillets marinated with different doses of hydrolates (1:1, 1:1.5, and 1:2) of *Lavandula luisiery*, *Thymus mastichina*, and *Origanum virens* during 1 and 9 days of storage.

Storage	Control			*Lavandula luisiery*										*Thymus mastichina*										*Origanum virens*										*p*-Value
(Days)				1:1			1:1.5			1:2			p-Dose	1:1			1:1.5			1:2			p-Dose	1:1			1:1.5			1:2			p-Dose	
CIE L*																																		
1	54.3	±	5.1	61.1	±	3.9	63.5	±	3.9	61.3	±	4.9	*ns*	61.8	±	2	62.7	±	3.1	62.4	±	2.1	ns	59.7	±	3.9	63.4	±	2.7	64.4	±	3.1	*ns*	*ns*
9	55.1 B	±	2.8	62.2 A	±	2.7	62.9 A	±	2.6	62.8 A	±	3.3	*ns*	63.0 A	±	3.4	62.5 A	±	2.8	63.2 A	±	3.1	ns	60.4 AB	±	2.7	61.9 A	±	2.3	63.5 A	±	3.8	*ns*	**
*p-storage*	ns			ns			ns			ns				ns			ns			ns				*ns*			*ns*			*ns*				
CIE a*																																		
1	3.5	±	2.9	1.9	±	1.6	1.7	±	1.2	2.5	±	1.8	*ns*	2.1	±	1	1.9	±	1.4	2.5	±	1.1	ns	2.4	±	1.2	2	±	0.8	2.5	±	0.6	*ns*	*ns*
9	4.7	±	1.8	3.3	±	1.1	3.2	±	0.5	3.8	±	1.6	*ns*	3.1	±	1.2	3.3	±	1.4	3.1	±	2	ns	4.3	±	0.9	3.2	±	0.5	4	±	1.8	*ns*	*ns*
*p-storage*	ns			ns			ns			ns				ns			ns			ns				*			ns			ns				
CIE b*																																		
1	13.5	±	1.4	12.6	±	0.6	12.5	±	0.3	12.1	±	0.2	*ns*	13	±	0.5	12.9	±	0.5	13.1	±	0.3	ns	12.8	±	0.5	12.9	±	0.5	12.8	±	0.4	*ns*	*ns*
9	14.6	±	0.7	14.4	±	1.2	14.4	±	0.2	15	±	0.4	*ns*	14.4	±	0.4	14.3	±	0.5	14.6	±	0.8	ns	15.2	±	0.7	14.5	±	0.4	15.5	±	1.5	*ns*	*ns*
*p-storage*	*			ns			***			***				*			*			*				**			*			ns				
Chroma																																		
1	14.2	±	1.8	12.9	±	0.9	12.7	±	0.4	12.5	±	0.4	*ns*	13.2	±	0.6	13.1	±	0.7	13.4	±	0.3	ns	13.0	±	0.7	13	±	0.5	13.0	±	0.3	*ns*	*ns*
9	15.4	±	0.9	14.8	±	1.3	14.8	±	0.2	15.5	±	0.7	*ns*	14.7	±	0.7	14.8	±	0.9	15	±	1	ns	15.8	±	0.9	14.9	±	0.5	16.1	±	1.9	*ns*	*ns*
*p-storage*	ns			ns			***			**				*			ns			ns				**			**			ns				
Hue																																		
1	76.6	±	11.3	81.8	±	6.1	82.6	±	5.4	78.7	±	8.1	*ns*	81.1	±	4.2	82.1	±	6	79.4	±	4.7	ns	79.7	±	4.9	81.4	±	3.9	79.1	±	2.7	*ns*	*ns*
9	72.5	±	6.7	77.3	±	3.6	77.5	±	2.1	75.8	±	5.7	*ns*	78.1	±	4.3	77.5	±	4.6	78.5	±	7.2	ns	74.2	±	2.6	77.7	±	1.8	76	±	5.1	*ns*	*ns*
*p-storage*	ns			ns			ns			ns				ns			ns			ns				*ns*			*ns*			*ns*				

Different uppercase letters in the same row indicate significant differences between groups (*p*-value). Significance levels: ns = not significant, * = *p* < 0.05, ** = *p* < 0.01, *** = *p* < 0.001.

**Table 5:** Oxidative status of fillets marinated with different doses of hydrolates (1:1, 1:1.5, and 1:2) of *Lavandula luisiery*, *Thymus mastichina*, and *Origanum virens* during 1 and 9 days of storage.

Storage	Control			*Lavandula luisiery*										*Thymus mastichina*										*Origanum virens*										*p*-Value
(Days)				1:1			1:1.5			1:2			p-Dose	1:1			1:1.5			1:2			p-Dose	1:1			1:1.5			1:2			p-Dose	
Lipid oxidation ^1^																																		
1	0.06 B	±	0.02	0.10 A	±	0.02	0.11 A	±	0.00	0.09 A	±	0.01	ns	0.06 B	±	0.02	0.05 B	±	0.00	0.05 B	±	0.01	*ns*	0.04 B	±	0.00	0.04 B	±	0.01	0.04 B	±	0.01	ns	***
9	0.08 B	±	0.01	0.10 A	±	0.01	0.10 A	±	0.01	0.10 A	±	0.01	ns	0.07 aBC	±	0.01	0.05 abCD	±	0.01	0.05 bCD	±	0.00	*	0.04 aDE	±	0.00	0.02 bE	±	0.00	0.04 aDE	±	0.00	**	***
*p-storage*	ns			ns			ns			ns				ns			ns			ns				ns			ns			ns				
Protein oxidation ^2^																																		
1	2.1 AB	±	0.1	2.1 AB	±	0.2	2.5 A	±	0.2	2.0 AB	±	0.3	ns	1.9 AB	±	0.3	1.9 AB	±	0.1	1.7 B	±	0.1	ns	1.9 AB	±	0.2	2.0 AB	±	0.6	2.0 AB	±	0.1	ns	*
9	2.3 AB	±	0.4	1.8 AB	±	0.4	2.3 AB	±	0.4	1.6 B	±	0.2	ns	2.1 AB	±	0.3	2.2 AB	±	0.3	2.4 AB	±	0.3	ns	2.5 A	±	0.1	2.4 AB	±	0.2	1.9 AB	±	0.5	ns	*
*p-storage*	ns			ns			ns			ns				ns			ns			ns				**			ns			ns				

Different lowercase letters in the same row indicate significant differences between doses for each hydrolate (*p*-Dose). Different uppercase letters in the same row indicate significant differences between groups (*p*-value). Significance levels: ns = not significant, * = *p* < 0.05, ** = *p* < 0.01, *** = *p* < 0.001 ^1^ mg MDA kg^−1 2^ nmol carbonyls mg protein^−1.^

**Table 6:** Two-ways ANOVA (hydrolate and storage effect) of the microbiological parameters, instrumental color and oxidative parameters of the marinated fillets with hydrolates.

	Hydrolate	Storage	Hydrolate × Storage
Microbiology			
Mesophilic	***	***	ns
Psychrophilic	***	***	ns
Total coliforms	***	*	ns
*E. coli*	ns	ns	ns
*S. aureus*	*	***	*
Instrumental color			
CIE L*	***	ns	ns
CIE a*	*	*	ns
CIE b*	*	***	ns
Chroma	**	***	ns
Hue	*	ns	ns
Oxidation			
Lipid oxidation	***	ns	ns
Protein oxidation	ns	ns	*

Significance levels: ns = not significant, * = *p* < 0.05, ** = *p* < 0.01, *** = *p* < 0.001.

## Data Availability

Dataset available on request from the authors.

## Supplementary Materials

The following supporting information can be downloaded at: https://www.mdpi.com/article/10.3390/antiox14121508/s1, Table S1: Volatile profile (percentage of total volatile compounds) of hydrolates.
