# Supplementary material for: Bioactivity of Aromatic Plant Hydrolates and Application for Pork Marination
Source: Antioxidants (Basel). 2025 Dec 16;14(12):1508. doi: 10.3390/antiox14121508 (PMC12729964; doi:10.3390/antiox14121508)
Supplement: Supplementary file 1 [file antioxidants-14-01508-s001.zip › antioxidants-4012231-supplementary.pdf]

Table S1. Volatile profile (percentage of total volatile compounds) of hydrolates.

Lavandula luisiery				*Thymus mastichina*				*Origanum virens*			
Compound (%)	Mean	±	SD	Compound (%)	Mean	±	SD	Compound (%)	Mean	±	SD
Eucalyptol	27.63	±	1.06	Eucalyptol	56.69	±	0.55	Thymol	93.57	±	0.09
α-Cyclogeraniol	15.56	±	0.62	α-Terpineol (*)	24.42	±	0.41	Carvacrol (*)	2.16	±	0.01
2-Cyclohexen-1-one, 3,4,4-trimethyl-	13.17	±	0.10	(-)-α-Terpineol	8.22	±	0.09	Borneol	1.75	±	0.03
Camphor	11.73	±	0.09	L-4-terpineol	5.49	±	0.12	4-Terpineol	0.85	±	0.01
2-Cyclopenten-1-one, 2,3,4,5-tetramethyl	7.16	±	0.25	β-Linalool (*)	3.03	±	0.07	1-Octen-3-ol	0.61	±	0.00
2-Cyclopenten-1-one, 3,4,5,5-tetramethyl-	6.98	±	0.06	trans-(-)-Pinocarveol	0.42	±	0.00	p-Cymen-8-ol	0.25	±	0.01
cis-Verbenone	5.11	±	0.38	(1R)-(+)-Norinone	0.35	±	0.03	α-Terpineol (*)	0.20	±	0.11
(1R)-(+)-Norinone	2.66	±	0.07	cis-Geraniol (*)	0.24	±	0.01	cis-3-Hexen-1-ol (*)	0.15	±	0.01
trans-(-)-Pinocarveol	1.93	±	0.02	Benzaldehyde (*)	0.19	±	0.00	Ethane, 1,1-dimethoxy-	0.08	±	0.01
Benzaldehyde (*)	1.44	±	0.17	Verbenone	0.18	±	0.01	trans-2-Hexenol (*)	0.08	±	0.01
Pinocarvone	1.26	±	0.03	cis-3-Hexen-1-ol (*)	0.15	±	0.08	Benzaldehyde (*)	0.05	±	0.00
(-)-Myrtenol	1.14	±	0.02	cis-Carveol	0.13	±	0.00	Eucalyptol	0.05	±	0.01
α-Isophorone	1.14	±	0.06	Pinocarvone	0.13	±	0.00	Butanal, 3-methyl-	0.04	±	0.00
1-Octen-3-ol	0.78	±	0.04	(-)-Camphor	0.12	±	0.00	Furfural	0.04	±	0.00
cis-3-Hexen-1-ol (*)	0.67	±	0.07	1-Octen-3-ol	0.07	±	0.00	Thymol acetate	0.03	±	0.01
3-Methyl-1-buten-3-ol	0.51	±	0.04	Elemol	0.07	±	0.00	Methyl 2-methylbutanoate	0.02	±	0.00
2-Butenal, 3-methyl	0.34	±	0.03	Cumyl alcohol	0.06	±	0.01	trans-Hex-2-enal	0.02	±	0.00
Furfural	0.32	±	0.01	β-Eudesmol	0.02	±	0.00	1,4-Cineol	0.02	±	0.00
2-Butanone, 3-methyl-	0.27	±	0.06	γ-Eudesmol	0.01	±	0.00	trans-(-)-Pinocarveol	0.01	±	0.00
Ethane, 1,1-dimethoxy-	0.20	±	0.01	Spathulenol	<0.01 (**)			m-Cymene	0.01	±	0.00

(*) Volatile compounds were identified by the analysis of commercial standards. SD: Standard deviation. (**) Detected in trace amounts but below the quantification limit (<0.01%).
